# Extracellular Domains of Transmembrane Proteins Defy the Expression Level–Evolutionary Rate Anticorrelation

**DOI:** 10.1093/gbe/evab235

**Published:** 2021-10-19

**Authors:** Chandra Sarkar, David Alvarez-Ponce

**Affiliations:** Department of Biology, University of Nevada, Reno, USA

**Keywords:** E–R anticorrelation, transmembrane proteins, misfolding avoidance hypothesis, translational robustness hypothesis

## Abstract

Highly expressed proteins tend to evolve slowly, a trend known as the expression level–rate of evolution (E–R) anticorrelation. Whereas the reasons for this anticorrelation remain unclear, the most influential hypotheses attribute it to highly expressed proteins being subjected to strong selective pressures to avoid misfolding and/or misinteraction. In accordance with these hypotheses, work in our laboratory has recently shown that extracellular (secreted) proteins lack an E–R anticorrelation (or exhibit a weaker than usual E–R anticorrelation). Extracellular proteins are folded inside the endoplasmic reticulum, where enhanced quality control of folding mechanisms exist, and function in the extracellular space, where misinteraction is unlikely to occur or to produce deleterious effects. Transmembrane proteins contain both intracellular domains (which are folded and function in the cytosol) and extracellular domains (which complete their folding in the endoplasmic reticulum and function in the extracellular space). We thus hypothesized that the extracellular domains of transmembrane proteins should exhibit a weaker E–R anticorrelation than their intracellular domains. Our analyses of human, *Saccharomyces* and *Arabidopsis* transmembrane proteins allowed us to confirm our hypothesis. Our results are in agreement with models attributing the E–R anticorrelation to the deleterious effects of misfolding and/or misinteraction.

Significance

Highly expressed proteins tend to evolve slowly, a trend known as the E–R anticorrelation and often attributed to them being under strong selection to not misfold or misinteract. However, the E–R anticorrelation is weaker or nonexistent among extracellular proteins, which could be due to the particular circumstances in which these proteins fold (the endoplasmic reticulum counts with mechanisms to deal with unfolded and misfolded proteins) or their extracellular location (which makes them unlikely to engage in misinteraction). We show that transmembrane proteins exhibit the usual E–R anticorrelation in their intracellular domains (which are folded and act in the cytosol), but not in their extracellular domains (which complete their folding in the endoplasmic reticulum and act in the extracellular space).

## Introduction

Proteins greatly differ in the paces at which they evolve: Whereas some proteins remain largely unaltered over long evolutionary periods, other proteins can quickly accumulate amino acid replacements in short periods of time (Zuckerkandl and Pauling 1965; Dickerson 1971; Li et al. 1985). One major factor affecting rates of protein evolution is gene expression: Highly expressed genes tend to encode slow-evolving proteins (Pál et al. 2001), a trend known as the expression–rate (E–R) anticorrelation. The reasons for this anticorrelation are, however, unclear (Pál et al. 2006; Alvarez-Ponce 2014; Zhang and Yang 2015).

A number of nonmutually exclusive hypotheses have been proposed to explain the E–R anticorrelation. The translational robustness hypothesis (Drummond et al. 2005; Wilke and Drummond 2006; Drummond and Wilke 2008) attributes the E–R anticorrelation to highly expressed proteins being under strong selective pressures to be able to fold properly despite the occurrence of translation errors. A significant fraction of proteins undergoes translation errors, which can lead to misfolding. The cytotoxic effects of protein misfolding are expected to be abundance-dependent. The misfolding avoidance hypothesis (Yang et al. 2010), an extension of the translational robustness hypothesis, proposes that highly expressed proteins are under increased selection to avoid misfolding (either due to mistranslation or to other factors). The misinteraction avoidance hypothesis proposes that highly expressed proteins are under stronger selective pressures to avoid undesired interaction with other proteins ( again, the negative effects of misinteraction are expected to be abundance-dependent; Levy et al. 2012; Yang et al. 2012 ). The mRNA folding requirement hypothesis proposes that highly expressed genes are under strong selection to exhibit highly stable folds, which in turn constrains protein evolution (Park et al. 2013). The function maintenance hypothesis proposes that proteins tend to be expressed at levels that optimize the tradeoff between the benefits of their function and the costs of synthesis (Cherry 2010; Gout et al. 2010).

Research in our laboratory has recently shown that secreted (extracellular) proteins lack an E–R anticorrelation (or in some species exhibit a weak E–R anticorrelation compared with nonsecreted proteins; Feyertag et al. 2017). This effect may be due to secreted proteins being less likely to undergo misfolding and/or misinteraction, and/or to such events causing less damage should they affect secreted proteins. First, secreted proteins are folded in the lumen of the endoplasmic reticulum, where a number of mechanisms known as the unfolded protein response prevent and deal with misfolded proteins (these mechanisms include chaperones and folding enzymes that recognize unfolded/misfolded proteins, and systems of quality control that sequester such proteins; Braakman and Hebert 2013). Second, secreted proteins act in the extracellular space, where misinteraction is less likely to occur and, should it occur, is expected to cause less damage. Thus, the translational robustness, misfolding avoidance, and misinteraction avoidance hypotheses are expected to apply less to secreted proteins than to nonsecreted proteins. In agreement with Feyertag et al.’s hypothesis that the lack of an E–R anticorrelation among secreted proteins was due to mitigation of misfolding, misinteraction and/or their deleterious effects, N-glycosylated proteins (a subset of secreted proteins that are subjected to very strict quality control) lack an E–R anticorrelation, and in fact exhibit a positive E–R correlation (Feyertag et al. 2019).

The results obtained by Feyertag et al. (2017) were robust to controlling for several differences between secreted and nonsecreted proteins. Nonetheless, it is conceivable that the lack of an E–R anticorrelation among secreted proteins might have been driven by some intrinsic characteristic of secreted proteins that we might have failed to control for. Transmembrane proteins are particularly interesting systems because they contain both intracellular domains (which are folded in the cytosol) and extracellular domains (which are folded, or at least complete their folding, inside the endoplasmic reticulum). Nascent transmembrane proteins are recruited to the outer surface of the endoplasmic reticulum, and some domains are translocated into the lumen of the endoplasmic reticulum as they are translated (White and von Heijne 2004; Skach 2009). We hypothesized that the extracellular domains of transmembrane proteins (similar to extracellular proteins) should lack an (or exhibit a weak) E–R anticorrelation, due to their exposure to the lumen of the endoplasmic reticulum during folding, and/or to the fact that they end up at the outer part of the cell membrane, where misinteraction and its deleterious effects are less likely. Conversely, intracellular domains of transmembrane proteins should exhibit the usual E–R anticorrelation, due to their synthesis and function in the cytosol (similar to intracellular proteins).

## Results

### Human Protein Abundances Correlate Better with the Rates of Evolution of Intracellular Domains

For each human gene, we identified the most likely mouse ortholog, aligned the encoded proteins, and used the resulting alignments to align the corresponding coding sequences (CDSs). We thus obtained a total of 16,581 human–mouse CDS alignments. For each alignment, we used the TMHMM server (version 2; Krogh et al. 2001) to predict the intracellular and extracellular domains. A total of 3,478 proteins were predicted to exhibit both kinds of domains and were thus inferred to be transmembrane proteins and retained for further analysis.

For each of these alignments, we estimated a separate nonsynonymous to synonymous divergence ratio (ω = *d*_N_/*d*_S_) for the intracellular and the extracellular fractions (which we called ω_i_ and ω_e_, respectively). As expected, ω_i_ and ω_e_ exhibited a positive correlation (Spearman’s rank correlation coefficient, ρ = 0.413, *P *=* *1.63 × 10^−143^). In addition, for more than half of the proteins, ω_e_ was higher than ω_i_ (1,813 cases; binomial test, *P *=* *0.013), consistent with the known high rates of evolution of extracellular domains (Heger et al. 2009). We binned proteins into three groups according to their protein abundances, and found that the percent of proteins for which ω_e_ was higher than ω_i_ was higher among proteins with high abundances (highly abundant proteins: 56%, intermediately abundant proteins: 52%, lowly abundant proteins: 51%).

**Fig. 1.**
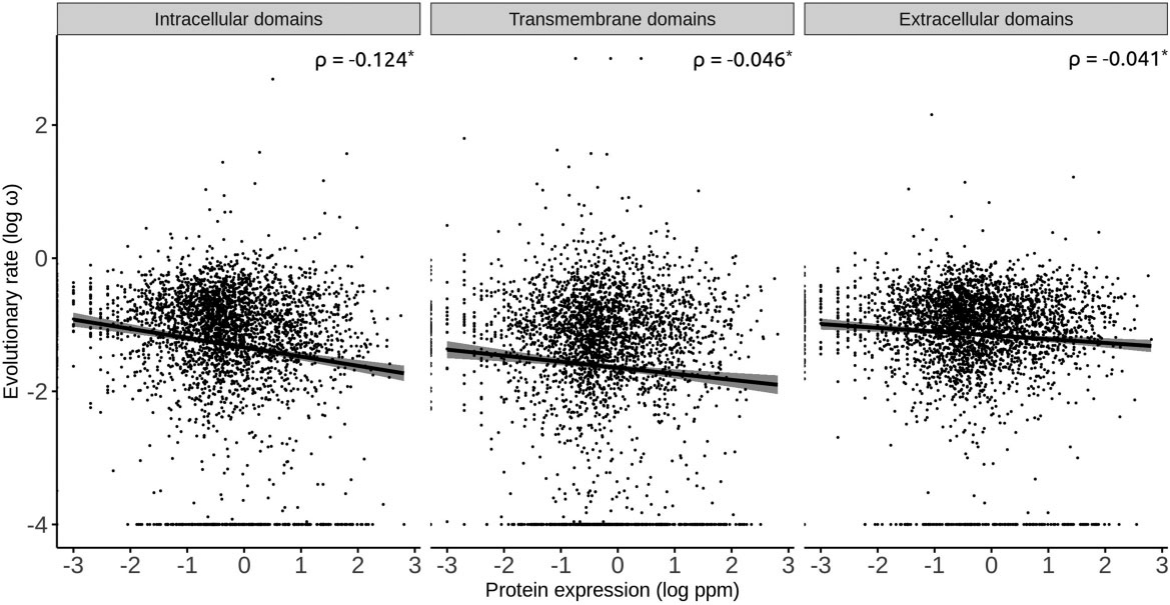
Correlation between rates of protein evolution and protein abundance in the intracellular, transmembrane and extracellular domains of human transmembrane proteins. *P <* *0.05.

Both ω_i_ and ω_e_ negatively correlated with whole-body protein abundances (fig. 1), but remarkably, the correlation was stronger for ω_i_ (ρ = −0.124, *n *=* *3,308, *P *=* *7.97 × 10^−13^) than for ω_e_ (ρ = −0.041, *n *=* *3,308, *P *=* *0.018). A Fisher’s *r*-to-*z* transformation test showed that the two correlation coefficients were significantly different (*Z* = −3.40, *P *=* *0.0003). Thus, as we had hypothesized, the E–R anticorrelation is stronger for intracellular domains than for extracellular domains.

We repeated our analyses using protein abundance data from 20 human tissues, with similar results. In all 20 cases, the correlation was more negative for ω_i_ (ρ ranged from −0.246 to 0.055) than for ω_e_ (ρ ranged from −0.187 to 0.166). The Fisher’s *r*-to-*z* transformation test found significant differences (ρ being significantly more negative for intracellular domains than for extracellular domains) in 10 of the tissues (supplementary table S1, Supplementary Material online).

### Human mRNA Abundances Correlate Better with the Rates of Evolution of Intracellular Domains

**Fig. 2.**
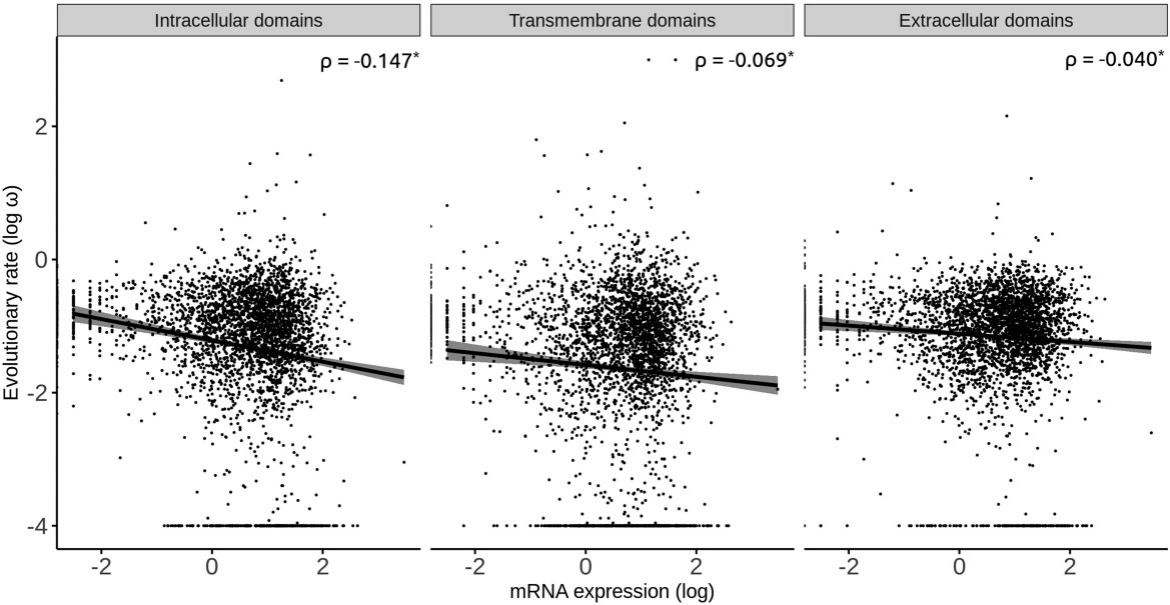
Correlation between rates of protein evolution and mRNA abundance in the intracellular, transmembrane and extracellular domains of human transmembrane proteins. *P <* *0.05.

For each human gene, we obtained mRNA abundance data for 32 tissues from the Human Atlas database (Uhlen et al. 2015) and computed the average across all tissues. The results were very similar to those for protein abundances: Average mRNA abundances correlate better with ω_i_ (ρ = −0.147, *n *=* *3,395, *P *<* *2.2 × 10^−16^) than with ω_e_ (ρ = −0.040, *n *=* *3,395, *P *=* *0.020), and both correlations were significantly different (*Z* = −4.44, *P *<* *0.0001) (fig. 2).

**Fig. 3.**
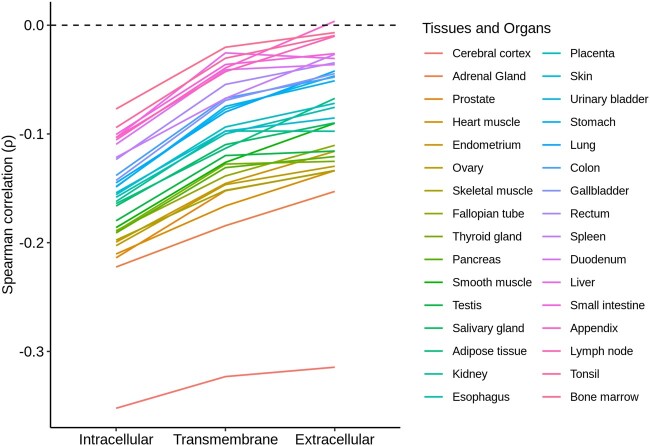
Correlation between rates of protein evolution and mRNA abundance in different tissues in the intracellular, transmembrane and extracellular domains of human transmembrane proteins.

We then analyzed the correlations between ω_i_ and ω_e_ and mRNA abundances in each of the 32 human tissues separately. In all 32 cases, the correlation was stronger for ω_i_ (ρ ranged from −0.352 to −0.077) than for ω_e_ (ρ ranged from −0.314 to 0.003) (fig. 3).

### Transmembrane Domains Exhibit an Intermediate E–R Anticorrelation

For each transmembrane protein, we estimated the nonsynonymous to synonymous divergence rate ratio of the transmembrane domains (ω_t_). As expected, ω_t_ positively correlates with both ω_i_ (ρ = 0.484, *P *<* *2.2 × 10^−16^) and ω_e_ (ρ = 0.498, *P *<* *2.2 × 10^−16^). For 1,646 of the proteins, ω_t_ was lower than both ω_i_ and ω_e_, a fraction that significantly exceeds one-third of the cases (binomial test, *P *<* *2.2 × 10^−16^); this is consistent with previous analyses showing that transmembrane domains tend to be highly constrained (Spielman and Wilke 2013).

The correlation between ω_t_ and protein abundance (ρ = −0.046, *P *=* *0.007) was intermediate between the ω_i_-protein abundance and ω_e_-protein abundance correlations (fig. 1). In 13 of the 20 human tissues analyzed, the correlation between ω_t_ and protein abundance was intermediate between the ω_i_-protein abundance and ω_e_-protein abundance correlations (supplementary table S1, Supplementary Material online); this ratio is significantly higher than one-third of the cases (binomial test, *P *=* *0.004).

Similarly, the correlation between ω_t_ and mRNA abundance (ρ = −0.069, *P *=* *4.33 × 10^−5^) was intermediate between the ω_i_-mRNA abundance and ω_e_-mRNA abundance correlations (fig. 2). In 30 of the 32 human tissues analyzed, the correlation between ω_t_ and mRNA abundance was intermediate between the ω_i_-mRNA abundance and ω_e_-mRNA abundance correlations (fig. 3); this ratio is significantly higher than one-third of the cases (binomial test, *P *=* *1.11 × 10^−12^).

### Consistent Results in Other Organisms

**Fig. 4.**
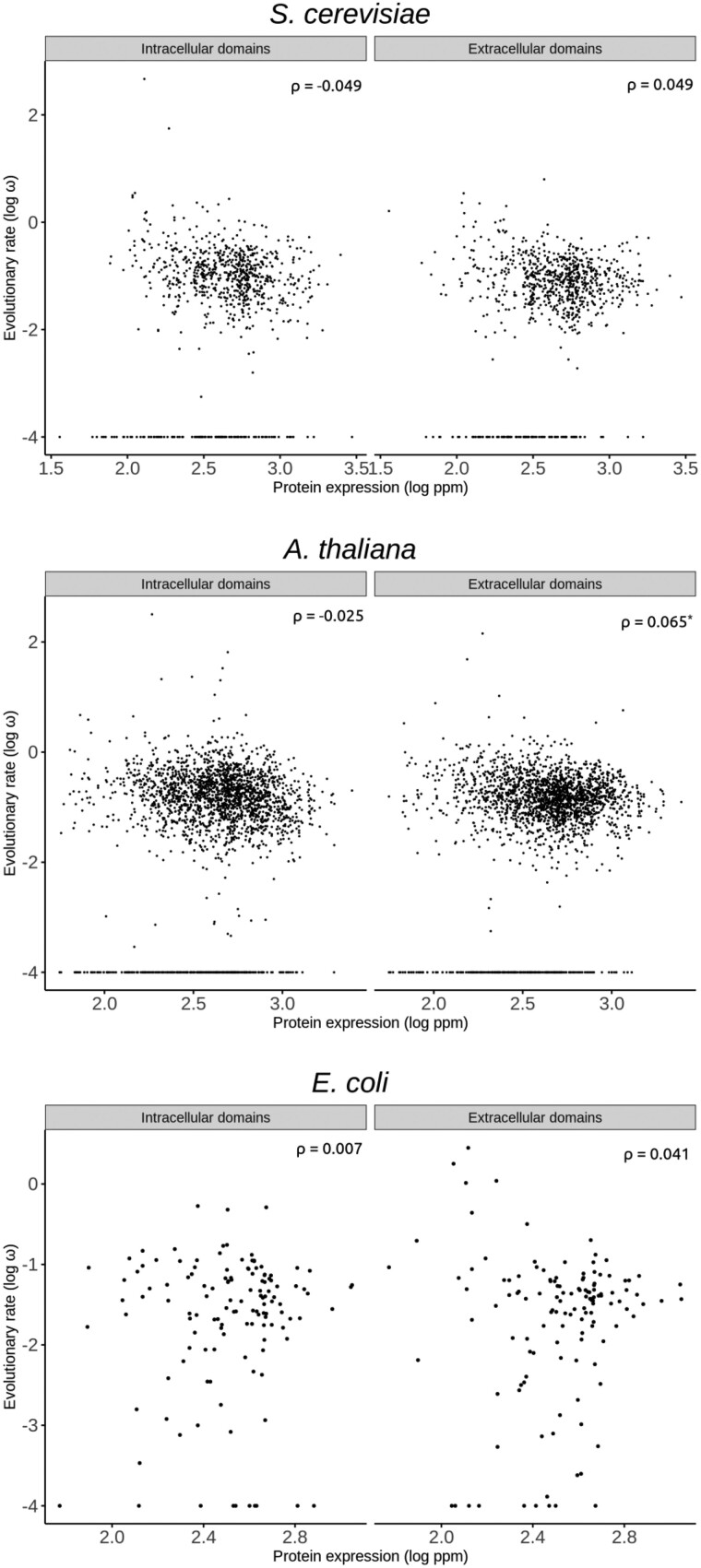
Correlation between rates of protein evolution and protein abundance in the intracellular and extracellular domains of transmembrane proteins of different organisms. *P <* *0.05.

To confirm whether the trend was specific to humans or, on the contrary, it could be observed in other, phylogenetically distant organisms, we analyzed pairs of *Saccharomyces cerevisiae*–*S. paradoxus*, *Arabidopsis thaliana*–*A. lyrata*, and *Escherichia coli*–*Salmonella enterica enterica* orthologs encoding transmembrane proteins. In all cases, the correlation between protein abundances and ω_i_ was more negative than that between protein abundances and ω_e_ (fig. 4).

For *Saccharomyces*, the E–R correlation was slightly negative for intracellular domains and slightly positive for extracellular domains, but nonsignificant in both cases (respectively, ρ = −0.049, *n *=* *829, *P *=* *0.157; ρ = 0.049, *n *=* *829, *P *=* *0.159). For *Arabidopsis*, the correlation was also slightly negative for intracellular domains and slightly positive for extracellular domains, in this case with a significant correlation for extracellular domains (respectively, ρ = −0.025, *n *=* *2,310, *P *=* *0.221; ρ = 0.055, *n *=* *2,310, *P *=* *0.002). For *Escherichia*/*Salmonella*, the correlation was close to zero for intracellular domains and slightly positive for intracellular domains, and nonsignificant in both cases (respectively, ρ = 0.006, *n *=* *130, *P *=* *0.942; ρ = 0.041, *n *=* *130, *P *=* *0.646). The Fisher *r*-to-*z* test was significant for *Saccharomyces* (*Z* = −2.00, *P *=* *0.023) and *Arabidopsis* (*Z* = −3.08, *P *=* *0.001), but not for *Escherichia/Salmonella* (*Z* = −0.27, *P *=* *0.394); we attribute the lack of a significant difference in *Escherichia/Salmonella* to the small number of transmembrane proteins available for analysis (*n *=* *130).

## Discussion

In summary, we have shown that protein and mRNA abundances correlate better with the *d*_N_/*d*_S_ values of intracellular domains (ω_i_ values) than with the *d*_N_/*d*_S_ values of extracellular domains (ω_e_ values) of human secreted proteins (figs. 1 and 2). The trend was consistently observed across mRNA abundance data of 32 human tissues (fig. 3). Similar results were also observed in three phylogenetically distant organisms (*Saccharomyces*, *Arabidopsis*, and *Escherichia*/*Salmonella*). Because both E–R correlations were computed on the same set of proteins, the different E–R anticorrelations that we observed cannot be a byproduct of any difference between the studied proteins.

These results are in agreement with our initial hypothesis that extracellular domains should exhibit an attenuated E–R anticorrelation, or no E–R correlation, due to the fact that they are folded in the lumen of the endoplasmic reticulum (where systems are in place to prevent and deal with misfolded proteins; Braakman and Hebert 2013), and/or because they end up acting at the outer part of the cell membrane (where misinteraction with other proteins is less likely to occur or to have deleterious effects). Indeed, some of the tenets of the translational robustness and misfolding avoidance hypotheses (namely, that a fraction of proteins misfold, with cytotoxic effects), and the misinteraction avoidance hypothesis (namely, that a fraction of proteins engages in undesired interactions with other proteins, also with cytotoxic effects) are expected to apply less to extracellular domains than to intracellular domains.

Our results are thus in agreement with the translational avoidance, the misfolding avoidance, and/or the misinteraction avoidance hypotheses (albeit they do not allow us to favor one over the others). However, our results would not be expected under the mRNA folding requirement or the function maintenance hypotheses alone, under which a similar E–R anticorrelation would be expected for extracellular and intracellular domains of transmembrane proteins. Nonetheless, it should be noted that our results do not rule out a relevant role of these hypotheses in partially explaining the E–R anticorrelation. For instance, mRNA folding has been shown to slow translation (thus increasing translational accuracy) at domains that are structurally important (Yang et al. 2014), which could affect the evolution of intracellular and extracellular domains differently.

Of note, the differences between the E–R anticorrelations of the intracellular and extracellular domains of transmembrane proteins (ρ = −0.124 and −0.041, respectively; fig. 1) are not as marked as the differences that Feyertag et al. (2017) observed between the E–R anticorrelations of intracellular and extracellular proteins (ρ = −0.259 and 0.038, respectively). The folding of extracellular domains is linked to that of the intracellular domains of transmembrane proteins (Houck and Cyr 2012); thus, extracellular domains may only partially benefit from the quality control mechanisms of the lumen of the endoplasmic reticulum, and/or these mechanisms may indirectly benefit intracellular domains, which would homogenize the E–R anticorrelations of intracellular and extracellular domains. However, at least another two factors may also be attenuating the differences between the E–R anticorrelations of intracellular and extracellular domains. The first are potential errors in the prediction of intracellular and extracellular domains: Some extracellular portions might have been erroneously predicted to be intracellular, and vice versa; this, however, seems unlikely given the high accuracy of the algorithm used (it correctly predicts 97–98% of transmembrane helices, and can discriminate intracellular and extracellular domains with specificity and accuracy above 99%; Krogh et al. 2001). The second possibility is that the *d*_N_/*d*_S_ estimates obtained in the current study, being based on smaller numbers of codons (only the intracellular or the extracellular ones), may be less accurate than those obtained by Feyertag et al. (2017) (based on full-length CDSs). In any case, we observe differences in the E–R anticorrelations of intracellular and extracellular domains, despite the potential confounding effect of these factors.

## Materials and Methods

Human and mouse protein and CDS sequences were obtained from the Ensembl database, release 62 (Cunningham et al. 2015). For each human gene, the longest protein/CDS was used. Human–mouse pairs of orthologs were identified using a best reciprocal hit approach (using BLASTP and *E*-value < 10^−10^). For each pair, protein sequences were aligned using ProbCons 1.12 (Do et al. 2005), and the resulting alignments were used to guide the alignment of the corresponding CDS sequences. The TMHMM server, version 2 (Krogh et al. 2001) was used to predict the intracellular and extracellular domains of each human and mouse protein. The results were used to separate each CDS alignment into an intracellular and an extracellular subalignment. Only proteins with both kinds of domains in both species were retained. PAML (version 4.4, model M0; Yang 2007) was used to estimate a separate *d*_N_/*d*_S_ ratio for each subalignment. Genes with *d*_S_ = 0 (and thus an infinite *d*_N_/*d*_S_ ratio) were removed. Equivalent analyses were conducted on pairs of *S. cerevisiae*–*S. paradoxus*, *A. thaliana*–*A. lyrata*, and *E. coli*– *Salmonellaenterica enterica* orthologs.

Protein abundances for human, *S. cerevisiae*, *A. thaliana*, and *E. coli* were retrieved from the PaxDB database, version 4 (integrated data sets were used; Wang et al. 2015). Messenger RNA abundances for 32 human tissues were obtained from the Human Atlas database, version 16.1 (Uhlen et al. 2015). For each gene, mRNA abundances were averaged across all tissues.

## Supplementary Material

Supplementary data are available at *Genome Biology and Evolution* online.

## Supplementary Material

evab235_Supplementary_Data

## Data Availability

All data used in this work are publicly available, as described in the Materials and Methods section.
