# Supplementary material for: Extracellular Domains of Transmembrane Proteins Defy the Expression Level–Evolutionary Rate Anticorrelation
Source: Genome Biol Evol. 2021 Oct 19;14(1):evab235. doi: 10.1093/gbe/evab235 (PMC8755491; doi:10.1093/gbe/evab235)
Supplement: evab235_Supplementary_Data [file evab235_supplementary_data.pdf]

Table S1. Correlation between rates of protein evolution and protein abundance in 20 human tissues

Tissue	*n*	Domains	Spearman's correlation		Fisher's *r*-to-*z* test	
			ρ	*P*-value	*Z*	*P*-value
Brain	1302	Intracellular	−0.246	$<2.2\times10^{-16}$*	−1.58	0.057
		Extracellular	−0.187	1.12×10^{-11}*		
		Transmembrane	−0.165	2.28×10^{-9}*		
Heart	1534	Intracellular	−0.090	0.0004*	−1.76	0.039*
		Extracellular	−0.026	0.3028		
		Transmembrane	−0.06	0.0187*		
Pancreas	1281	Intracellular	−0.078	0.0054*	−2.58	0.005*
		Extracellular	0.024	0.3826		
		Transmembrane	−0.007	0.7995		
Saliva	194	Intracellular	0.051	0.4833	−1.12	0.131
		Extracellular	0.164	0.0227*		
		Transmembrane	0.253	0.0003*		
Skin	336	Intracellular	−0.113	0.0381*	−1.56	0.059
		Extracellular	0.007	0.8944		
		Transmembrane	−0.016	0.7748		
Placenta	904	Intracellular	−0.135	4.60×10^{-5}*	−2.13	0.017*
		Extracellular	−0.036	0.2846		
		Transmembrane	−0.065	0.0496		
Colon	1033	Intracellular	−0.100	0.0012*	−2.05	0.020*
		Extracellular	−0.010	0.7366		
		Transmembrane	−0.009	0.7755		
Plasma	594	Intracellular	0.055	0.1824	−1.94	0.026*
		Extracellular	0.166	4.74×10^{-5}*		
		Transmembrane	0.037	0.3716		
Testis	1803	Intracellular	−0.141	1.93×10^{-9}*	−1.59	0.056
		Extracellular	−0.089	0.0002*		
		Transmembrane	−0.035	0.1387		
Esophagus	708	Intracellular	−0.060	0.1131	−2.23	0.013*
		Extracellular	0.059	0.1168		
		Transmembrane	0.001	0.9874		
Kidney	976	Intracellular	−0.025	0.4300	−1.51	0.066
		Extracellular	0.043	0.1767		
		Transmembrane	0.015	0.6463		
Platelet	844	Intracellular	−0.031	0.3641	−0.50	0.309
		Extracellular	−0.007	0.8358		

		Transmembrane	0.005	0.8840		
Urine	523	Intracellular	−0.025	0.5692	−2.94	0.002*
		Extracellular	0.156	0.0003*		
		Transmembrane	0.026	0.5581		
Female gonad	1665	Intracellular	−0.138	1.39×10^{-8}*	−2.36	0.009*
		Extracellular	−0.057	0.0194		
		Transmembrane	−0.034	0.1635		
Liver	2164	Intracellular	−0.037	0.0814	−2.12	0.017*
		Extracellular	0.027	0.2102		
		Transmembrane	−0.005	0.8256		
Prostate	1210	Intracellular	−0.080	0.0052*	−1.30	0.097
		Extracellular	−0.027	0.3402		
		Transmembrane	−0.034	0.2363		
Uterus	402	Intracellular	−0.054	0.2826	−1.13	0.129
		Extracellular	0.026	0.5996		
		Transmembrane	0.016	0.7554		
Gallbladder	942	Intracellular	−0.036	0.2740	−1.81	0.035
		Extracellular	0.048	0.1432		
		Transmembrane	−0.023	0.4761		
Lung	919	Intracellular	−0.063	0.0559	−1.60	0.055
		Extracellular	0.011	0.7287		
		Transmembrane	−0.025	0.4550		
Rectum	1072	Intracellular	−0.105	0.0006*	−2.14	0.016*
		Extracellular	−0.013	0.6707		
		Transmembrane	−0.025	0.4115		

*, $P < 0.05$.
